# Spouses of Military Members' Experiences and Insights: Qualitative Analysis of Responses to an Open-Ended Question in a Survey of Health and Wellbeing

**DOI:** 10.1371/journal.pone.0114755

**Published:** 2014-12-05

**Authors:** Catherine E. Runge, Michael Waller, Alison MacKenzie, Annabel C. L. McGuire

**Affiliations:** The University of Queensland, School of Population Health, Centre for Australian Military and Veterans' Health, Queensland, Australia; University of Texas Health Science Center at San Antonio, Research Imaging Institute, United States of America

## Abstract

**Introduction:**

There are few studies on the experiences of spouses of military members, with most focused on adverse impacts of deployment. Responses to an open-ended question in a survey of spouses' health and wellbeing enabled access to perceptions and insights on a broad range of topics. The objective of this investigation was to examine how respondents used the open-ended question and what they discussed, in aim of informing support service agencies and spouses of military members.

**Methods:**

Thematic analysis was conducted on responses to the open-ended question. Descriptive analysis was performed on the demographics, military member characteristics and self-reported health of respondents and non-respondents to the open-ended question.

**Findings:**

Over a quarter (28.5%) of the 1,332 survey participants answered the open-ended question, with respondents having a significantly higher level of education than non–respondents. Respondents expressed negative and positive experiences and insights on military life, provided personal information, commented on the survey, and qualified their responses to closed-ended questions. Topics included ‘inadequate support’, ‘deployment impacts’, ‘suggestions for supporting agencies’, ‘appraisal of experiences’ and ‘coping strategies’.

**Conclusions:**

This investigation uncovered issues of importance to spouses of military members that were not included or identified in a quantitative study. The findings provide a platform from which to explore these issues further, particularly the impact of military life on the non-serving spouse's career. The findings also provide support agencies with evidence to strengthen their services and they give spouses an opportunity to reflect on their own and others' feelings and evaluations of military life.

## Introduction

There is increasing recognition of the role that families play in the recruitment, effectiveness and retention of military members [1]. Most research on spouses of military members is quantitative and focused on the impact of the military member's deployment on their spouse's psychological health [2]. Numerous adverse outcomes have been identified, such as lower mental and physical wellbeing, depression and reduced relationship satisfaction [3]–[5].

Qualitative research on spouses of military members has most commonly used individual interviews to examine facets of military life. Specific to deployment, spouses have endorsed family, community and military–focused support, support drawn from children, gathering information and preoccupation as coping strategies [6]–[9]. Worry, loneliness, assuming dual roles, renegotiating roles and relationships, and recognising strength have been described as key characteristics of the deployment experience [6], [8], [10]. For military life in general, spouses associated the characteristics of being realistic and flexible with successful adjustment to military life and endorsed continued self-development as advice for new spouses [11]. On the issue of spouse employment, interviews with over one thousand spouses of military members revealed that almost two-thirds believed that the military negatively impacted their own employment [12].

Two qualitative analyses of Australian military spouses have been reported. Interviews with 76 spouses aimed to increase understanding of what it means to be supported through a deployment. Spouses wanted and expected Defence agencies to provide support calls during separation, felt understood by others going through similar experiences and renegotiated family roles using previous experience, intuition and education [13]. A survey of spouses of military members' evaluations of the Australian Defence Force included one open-ended question on stressors related to the absence of the military member and one on Defence support for families. The most common theme for stressors was dealing with everyday demands alone without the support of the military member [1]. On Defence support, responses revealed perceptions that support had improved, families had to proactively access support and feeling supported often depended on unit-level leadership.

The survey study from which the present investigation is drawn found that spouses of military members who have experienced deployment were in the normal range for physical and mental health and wellbeing [14]. Additionally, most partners felt supported and positive about their relationship with the military member and reported moderate to very high levels of family satisfaction. Segal contends in her seminal paper that the military and the family, more so than other societal institutions, are ‘greedy institutions' that make great demands on time and loyalty [15]. While the survey provided evidence that most families were doing well, it could not determine how families negotiated between these two institutions. A broad open-ended question was included at the end of the survey to capture such evidence. The present investigation is a qualitative analysis of the responses to this question.

Many researchers collect information from concluding open-ended questions in surveys but fail to analyse or present the replies to these questions [16], [17]. The rationale to include a concluding open-ended question in a survey is to: provide illustration and understanding of responses to closed-ended questions; identify issues of importance to respondents not covered in the survey; obtain feedback on the research process; and inform the content of further surveys [16], [18]. While there can be a negativity bias in open-ended question responses [18], the potential to achieve a large number of responses and diversity of topics provides researchers with rich data. They have demonstrated usefulness for: obtaining perceptions and concerns [17], [19]; the research process [20]; and informing practice [18].

Understanding the perceptions of spouses of military members on their own health and wellbeing is beneficial for research, supporting agencies and other spouses. The objective of the present investigation was to provide an overview of how respondents replied to a broad open-ended question and what they discussed, in aim of providing such benefits.

## Methods

The data is drawn from a cross-sectional survey on family health and wellbeing that was completed by spouses of Australian Defence Force members. The survey was administered by researchers at the then Centre for Military and Veterans' Health at The University of Queensland in 2011 as part of the Timor-Leste Family Study. The study methods are described in detail in the study report [14]. In summary, a sample of spouses of Navy, Army and Air Force regular and reserve members, who were serving or ex-serving at the time of the study, were invited to complete a survey that comprised questions on: demographics; the military member's deployments; physical and mental health; family functioning; work/family conflict; relationship satisfaction; intimate partner violence; coping styles; social support; use of support services; barriers to seeking care; attitudes to the military; and the emotional and behavioural health of their children. The survey concluded with the open-ended question: ‘Is there anything else which you feel is relevant to this study that you would like to tell us about?’ The inclusion of the question was not theoretically driven. The responses were analysed using thematic analysis to build a picture of the respondents' collective experiences [21].

Due to the broad question and the manageable number of responses (n = 379), manual coding of the data was chosen over computer-assisted analysis. Analysis occurred at two levels – first to identify how respondents used the question (type of response), for example, to comment on the survey, and second to identify what they discussed (topic of response), for example, the length of the survey. Each author read the entire dataset and individually assigned a short descriptor for type of response to the same subset of 75 responses. A codebook was developed from the consensus on the range and wording of descriptors for type of response. The authors used the codebook to code the remaining responses (76 responses each and an additional 10 responses assigned to another author). Discrepancies between authors were resolved through discussion and majority consensus. The same process was used to code the topic of response. Types and topics of responses that were anticipated based on the literature and those that were not anticipated were identified [22]. Through an iterative process the number of topics was consolidated. Quotations are used for illustration and they were edited to protect identities, correct spelling, omit extraneous words, and to provide explanatory information.

Descriptive analysis examined characteristics of respondents and non–respondents using chi-squared tests of association. The comparison was limited to non–respondents who had completed the survey section that preceded the open-ended question to counter potential overestimation of non-response.

### Ethics statement

The investigation was approved by: the Australian Department of Veterans' Affairs Human Research Ethics Committee (E010/002); the Australian Defence Human Research Ethics Committee (578-10); and the University of Queensland Behavioural and Social Sciences Ethical Review Committee (2010000621). All invitees received an information sheet that described the study and the rationale for its conduct. All participants provided written informed consent and were provided with contact details for support services.

### Findings

Over a quarter (28.5%, n = 379) of the 1,332 survey participants answered the open-ended question. Table 1 shows the characteristics of respondents to the open-ended question. Respondents have a statistically significant higher level of education than non–respondents. Older spouses responded more frequently than younger spouses to the open-ended question.

**Table 1:** Characteristics of respondents and non-respondents to the open-ended question.

	Respondents (n = 379)		Non-respondents (n = 1,120)		
	n	(%)	n	(%)	p-value*
**Sex**					
Female	345	(34.5)	655	(65.5)	
Male	34	(28.3)	86	(71.7)	0.18
**Age**					
18–29	31	(31.6)	67	(68.4)	
30–39	123	(32.5)	256	(67.5)	
40–49	128	(32.2)	269	(67.8)	
50+	94	(42.0)	130	(58.0)	0.06
Not reported	3		19		
**Education**					
Year 10 or below (Jnr-Mid High School)	29	(24.8)	88	(75.2)	
Year 11 or 12 (Snr High School)	50	(25.0)	150	(75.0)	
Certificate or diploma	132	(33.8)	259	(66.2)	
Bachelor degree or above	167	(41.2)	238	(58.8)	<0.0001
Not reported	1		6		
**Employment**					
Full-time	160	(33.3)	321	(66.7)	
Part-time/casual	124	(37.6)	206	(62.4)	
None	93	(32.0)	198	(68.0)	0.29
Not reported	2		16		
**Spouse's own military status**					
Serving or ex-serving	93	(31.7)	200	(68.3)	
Never served	260	(34.9)	485	(65.1)	0.33
Not reported	26		56		
**Family composition**					
Children living with the spouse	264	(32.8)	540	(67.2)	
No children living with the spouse	100	(36.8)	172	(63.2)	0.24
Not reported	15		29		
**Military member's service type**					
Active Regular	251	(34.3)	481	(65.7)	
Active Reserve	46	(34.8)	86	(65.2)	
Ex-serving/Inactive Reserve	82	(32.0)	174	(68.0)	0.78
**Military member's Service**					
Navy	75	(34.1)	145	(65.9)	
Army	230	(34.1)	425	(64.9)	
Air Force	74	(30.2)	171	(69.8)	0.38
**Length of relationship with military member**					
0–4 years	20	(30.8)	45	(69.2)	
5–9 years	78	(33.8)	153	(66.2)	
10–19 years	121	(30.0)	282	(70.0)	
20–29 years	99	(38.5)	158	(61.5)	
30+ years	59	(39.3)	91	(60.7)	0.12
Not reported	2		12		
**General Health (SF-12, Question 1)**					
Excellent/Very good	198	(31.9)	424	(68.2)	
Good	129	(35.2)	237	(64.8)	
Fair/Poor	50	(38.5)	80	(61.5)	0.26
Not reported	2		0		

*Chi-square test of association.

The authors identified six types of response and 26 topics of response. The median response contained 69 words (minimum 4, maximum 1,374). Three types of response were about experiences and insight, two types reflected on the survey and the other was the provision of personal contextual information. Half of all responses were not exclusive to one type of response. Table 2 shows an overview of the findings, with response frequencies reported to provide a broad indication of the topics of importance to this group of spouses of military members. Two specific issues discussed by four respondents did not fit into a type of response and these are shown in Table 2.

**Table 2:** Type and topic of response to the open-ended question and example responses.

Type and topic of response		
Number of responses	n	Example responses
**Negative experiences of military life**		
Inadequate support	62	I was extremely disappointed about the support for me while he was away (R111)
Deployment impacts	55	Dad having 5 deployments in 9 years resulted in most of the children's worries (R16)
Posting impacts	41	We have hardly had a single move that has not had various housing problems (R110)
Separation impacts	31	I suffer from anxiety–it definitely increases when my husband goes away (R203)
Spouse employment impacts	26	Each time [posting] I have had to make the sacrifice of giving up a job I love (R102)
Defence demands on members	21	We love the Defence Force but they are breaking their senior members (R206)
Transition to civilian life	14	Physically and mentally members are not prepared for the outside work force (R27)
Inadequate pay/entitlements	5	There is still an inequality with regards to pensions on retirement (R175)
**Insights on military life**		
Suggestions for support agencies	55	Giving military personnel positive parenting examples would be a good step (R84)
Appraisal of experiences	49	Years ago I would have listed ADF work as an issue–current posting is fantastic (R68)
Coping strategies	35	We are optimists and will always make the most of situations (R75)
Decision to leave service	9	We left the ADF for financial reasons but wonder if we've done the right thing (R171)
Recognising organisational change	8	The balance between work/family is slowly being addressed within Defence (R67)
Civilian attitudes	7	Civilian friends/family never understand what hardship you are going through (R208)
**Personal information**		
Military member information	41	My husband is a member of the Specialist Reserve (R332)
Child information	40	My child's difficulties are mainly due to her having Down Syndrome (R243)
Dual-serving/ex–serving families	31	My wife deployed to Rwanda and I deployed to East Timor and Iraq (R184)
Respondent information	15	I am currently the primary carer for my father-in-law who has Alzheimer's (R169)
**Comments on the survey**		
Survey content, design & timing	31	Survey seemed to focus on the "troublesome" side of being a service spouse (R268)
Suggestions for further studies	19	PTSD of Defence members and effects on family. Alcohol & drug abuse (R120)
Study appreciation and follow-up	9	Thank you for the opportunity to undertake this survey–we really appreciate it (R379)
**Positive experiences of military life**		
Good support	20	Support agencies were available and effective. Nil problems experienced (R352)
Growth/opportunities/satisfaction	18	My children developed the ability to make friends easily (R303)
Pride or support in military service	12	I have always really valued the role of the ADF–its personnel and their families (R153)
Good pay/entitlements	4	Deployments have given us financial advantages we would never have had (R129)
**Closed-question qualifications**	45	This questionnaire would be filled out differently if my husband was away (R198)
**Specific issues**		
Military member exposures	2	The only concern I have is the amount of medications that were given to them (R242)
Medical confidentiality	2	Case where medical records were taken by Defence lawyers is of grave concern (R18)

### Negative experiences of military life

#### Inadequate support

Responses that conveyed perceptions of inadequate organisational support for spouses and families were the most frequent response. Many responses related to the spouse expecting contact from Defence agencies during deployment and feeling disappointed when this expectation was not met:

Not a single Army person called me to see how I was going while my husband was in Afghanistan for 10 months and I was at home looking after our baby (R122).

Spouses expressed poor quality support for families without children, families with older children, male spouses and spouses of senior and reserve members, often indicating that support was mostly tailored for families with young children:

During [husband's] second deployment I received a colouring book designed for pre-schoolers that my 15–22 [year olds] thought a hoot [amusing] (R145).

Some respondents challenged assertions that Defence is ‘family friendly’, citing excessive work demands and describing unsupportive attitudes from particular personnel:

I felt a lot of time was wasted at work that could be better spent with families. I felt that my husband's superiors were not really all that interested about the family situation (R10).

Organisational support for the military member during transition from member to veteran caused concern, particularly when medical or mental health issues were involved. The process of applying for Veterans' Affairs compensation also caused grief for some families.

#### Deployment impacts

Spouses discussed the impacts of the military member's deployment/s on their family's wellbeing. They described transient impacts and impacts that were ongoing. Ongoing impacts were mostly related to mental health issues:

When he came home [from deployment] he was very quiet and could not relate to myself or our children. This went on for about a month and then everything was OK (R273).

My partner was diagnosed with PTSD [posttraumatic stress disorder] following Timor [deployment]. Unfortunately we still battle depression type 'episodes' (R55).

Stress from sole-parenting and worrying about the military member was the main impact of deployment on spouse health:

I looked back and realised that for the last 3 months of his deployment I couldn't seem to get well, as I think that I was so stressed, but you have to keep going (R60).

More spouses discussed adverse impacts for their children than for themselves. They explained that deployment impaired the child's relationship with their deployed parent or resulted in psychological and social issues for their child:

7 year old son developed severe anxiety about being alone in his home when Dad deployed to Iraq (R74).

Multiple and extended deployments were described as particularly difficult for the family, as was short notice of deployment and deployments that occurred not long after moving to a new location.

#### Posting impacts

Postings are the movement to a new location every two to four years to meet Defence requirements. The family of a military member can choose to move with the military member or stay in their current location. Expressions of the challenges postings present for spouse employment were the most common topic of response, however, because spouse employment was also raised in general it is presented as a separate topic.

Aside from spouse employment, responses focussed on adverse impacts on establishing support networks and forming long-term friendships, missing extended family, impacts on child education and adjustment and problems with housing and the moving process. Some spouses noted that the impact of postings on maintaining a supportive network accumulated:

The anxiety I feel in not knowing where to make a base for our family is becoming increasingly acute. The idea of starting all over again with people you have no history with becomes more difficult each time [new posting] (R30).

Regarding child education and adjustment, the lack of a national curriculum and moving older children were common concerns:

When they were younger it was easier, pack up and go, but as they matured – new schools…making new friends, was less than appealing and increasingly distressing for them (R334).

#### Separation impacts

Separation from the military member occurs for reasons other than deployment. The member may post away from their family and training and exercises (training for deployments) can take them away for weeks or months. Some spouses explained that these types of separations often had a greater impact on the family than deployment.

Adverse impacts of separation for children comprised emotional reactions and some psychological manifestations. Like deployment impacts, separation also impaired some children's relationship with the military parent:

My husband has been away all year training. My 7 year old son was teary and emotional during this lengthy period of absence of Dad (R106).

My 2 boys have suffered by not having a close relationship with their father, (due to him being absent so much) and in the case of one son I feel it has contributed to a drug problem and a severe fracturing of family dynamics (193).

Impacts for spouses centred on the challenges of sole parenting, maintaining the home front and the spousal relationship and lamentations that the military member missed their children growing up and family events:

My husband has not been at home for more than 1–2 months at a time for 6 years…very straining on relationship (R168).

#### Spouse employment impacts

Spouses discussed the impact of the posting cycle or the demands of the military member's service on their own employment and careers. Having to find new jobs, accept jobs at a lower level and curtailing personal ambitions caused frustration:

Just when you start to prove yourself in a company and gain credibility and wages increase you are up for a posting again and finding yourself at entry level again (R285).

Decreased emotional wellbeing and financial hardship were described as the key impacts of lost or changed employment. Some spouses explained that this situation was part of marriage to a military member and they either accepted it or endured it. Others felt that the Defence Force did not take spouse careers into consideration when posting members or provide members with the flexibility to adequately attend to family roles:

Being a RAAF [Royal Australian Air Force] wife has dramatically affected my work prospects. This is just part and parcel of being a RAAF wife (R377).

It's going to be me again that has to quit my job because I can't juggle career and family without his [husband] help. Army doesn't care about sharing family responsibility or spousal career demands (R3).

#### Defence demands on members, transition to civilian life and inadequate pay and entitlements

Impacts on the family from the military member's workload were discussed. Responses encapsulated expressions of high workloads, a pressure to attend to duties outside of work hours and frustration at ‘last minute’ commitments or changes to commitments. Spouses also noted adverse impacts of ‘additional’ responsibilities of military members:

After [deployment], work commitments that are unimportant to families (e.g. marching for the community) are always forced on the family and these cause more stress at a time when you're trying to renegotiate a relationship (R38).

Spouses of ex-serving military members relayed difficulties associated with the member leaving the Defence Force. They noted that ex–serving members struggled with knowing what direction to take following service:

[Husband] has tried different activities [after leaving service]…the last resulted in almost destroying our marriage. I think he is now accepting the fact that his working life is not what he thought it was going to be (R124).

A small number of spouses discussed inadequate military pay and military and veteran entitlements.

### Insights on military life

#### Suggestions for support agencies

Spouses discussed ways that supporting agencies could improve the military life experience of families. Some pointed out areas that need improvement and others provided specific ideas. Most commonly they argued for targeted support for different types of families at different stages of life, such as advising organisations that they “need more support for military wives that do not have children” (R91). Some spouses reflected on support that they considered effective and several highlighted that there is a range of support, but it is not actively sought out by spouses:

I believe that Defence offers a number and varied options to assist partners if one wishes to seek help. Some don't think to, and this is where unit, regiments, ships etc. need to be considerate, e.g. I recall a register [regiment name] used once a month to call partners and it worked very well (R140).

Other responses discussed support for the military member. Several spouses expressed a desire for involvement in the military member's health consultations and for longer psychological follow-up of members post-deployment:

I strongly believe that Defence psychologists should have contact with spouses (or family) in reference to post-deployment checks (R14).

#### Appraisal of experiences

These responses were mostly provided by spouses who clearly had long experiences of military life and focused on how experiences changed over time and circumstance. Spouses explained the decisions their family had made regarding military and family life and many noted that their life stage influenced their experiences:

Now in my late 40's the "military life" is easier for me now (R288).

They also pointed out that their own and their family's personalities and the quality of their relationships often had much greater influences on their wellbeing than influences from military life. Supportive relationships were central to positive evaluations of military and family life:

Our whole family life has revolved around the Defence life. It has had both a positive and negative effect on our family but as a whole our family is strong and supportive (R179).

#### Coping strategies

Spouses discussed strategies they used to meet military life challenges, particularly deployment. Personal strategies focused on developing routines for the family and practical strategies comprised accessing the support available:

Second time around [subsequent deployment] we used allowances to pay for a yard-worker & some cleaning to lift the load a little and it all went much smoother (R365).

A number of spouses were aware that their coping abilities influenced how their children experienced deployment and ‘making the most' of a situation was a good strategy. They also noted that they developed their coping strategies over time and recognised that spouses with less military life experience can have difficulties. Longer experience with military life also enabled spouses to learn what support was available and effective for them:

With each subsequent deployment I was more familiar with the type of support that I could access and endeavoured to use this support to keep myself informed and reasonably happy (R235).

Responses that discussed the value of family and social support and sharing experiences with others going through similar experiences (particularly deployment) were common:

I am supporting younger wives…this I feel is helping them and allows me to pass on some 'lessons learnt'. I remain in contact with an 'army wife' from the Vietnam [War] era – they have been supportive to me over the years (R380).

#### Decision to leave service, recognising organisational change and civilian attitudes

Spouses explained the reasons why their family decided to leave service or will be leaving service. The reasons comprised missing the military member, the member's excessive workload, wishing to secure educational and social stability for their children and finances. Recognition of improved conditions in the Defence Force stopped some families from leaving service:

Defence is slowly adopting flexible work practices. This is the sole factor that has encouraged us to remain (R221).

A small number of spouses commented on ill-informed civilian attitudes to military life.

### Positive experiences of military life

#### Good support

Spouses praised organisations associated with the Australian Defence Force for the support they provided. The Defence School Transition Aides (who provide in-school support for children of military members) were particularly singled out:

They provide extra support to our children which is invaluable and I hope this role continues (R69).

In contrast to the inadequate support during deployment responses, several spouses acknowledged that they received good deployment support:

The information and assistance provided by the ADF [Australian Defence Force] before my partner's deployment to East Timor was very good (R251).

The Australian Department of Veterans' Affairs were specifically praised for counselling services:

DVA [Department of Veterans' Affairs] was excellent in providing me with easy access to funding for private counselling sessions with a psychologist of my choice (R217).

#### Growth, opportunities and satisfaction

Personal and family growth and good opportunities were described as positives of military life. The notion of ‘becoming stronger' from military life was particularly communicated. Spouses also discussed the opportunities military life provided, particularly for travel and making friends around the country, and a few explained that the military member's job satisfaction flowed onto the family:

I developed a strong personality of my own…Our girls say that as a consequence they have learned to be liberated independent women in their own right too (R358).

I am happy for my partner to go away on Defence work because I know he will return in a happy state of mind, having spent time with his friends doing something he enjoys (R145).

#### Pride or support in military service and good pay and entitlements

Spouses expressed pride or support in the military member's service and in the military in general and a small number discussed good military pay and entitlements.

### Comments on the survey

#### Survey content, design and timing

The survey content and its applicability to all participants prompted comment. The use of mental health measures, with items such as “about how often did you feel hopeless”, resulted in several spouses feeling that the survey only examined adverse outcomes:

You haven't asked me what we do that has made 34 years of marriage and family work. Nor have you asked how it could have been better (R353).

Other spouses felt that the survey was not particularly applicable to them for a range of reasons, such as they were also a military member who had deployed. Comments on design were that the survey was too long or certain questions could be answered in different ways depending on interpretation. A number of spouses questioned the study timing – they felt it should have occurred closer to the time of the Timor-Leste deployments (the bulk of personnel deployed in 1999–2000) or just longer ago:

This study should have been done years ago, to prevent all the strain on families (R71).

#### Suggestions for further studies

Spouses discussed issues they felt should be studied. A number suggested that capturing how much time the military member is away from home is more important than looking at deployment in isolation:

My partner has had to do many long stints away for other reasons – while this is important for his role – they also have the same effect on families [as deployment] (R244).

Issues that spouses expected on the survey or would like to be explored in further studies comprised: spouse employment; effects of posttraumatic stress disorder on families; children with special needs; female military members; and impotence. For exploring deployment, spouses wanted more specific questions about the experience, such as the impact of the ‘reunion’ stage:

Nothing seems to be asked about the 6 months after their return [from deployment]; everyone I know thinks this period is more stressful then when they are gone, especially with children (R157).

#### Study appreciation and follow-up

While many spouses ended their response with “thank you”, nine spouses explicitly expressed appreciation for the study or the opportunity to express themselves. Four of these spouses offered the study team further contact to discuss their response or conduct an interview.

### Personal information

Personal information about the spouse and their family that could not be captured in the closed-ended questions was provided in the open-ended question. Information about the military member comprised health conditions, their personality and most commonly, current situations such as postings and deployments. The survey did not contain questions about child physical health and therefore a number of spouses shared such information, particularly those with children with special needs. A small number of spouses provided information about their own health or personal situation.

### Closed-question response qualifications

Spouses also used the open-ended question to give reasons for their closed-ended question responses. Most often they explained that their current separation from the military member influenced their closed-ended question responses, while others explained that they answered the survey based on past experiences. A number explained that their current situation, such as pregnancy or a current stressful event accounted for some of their feelings or symptoms.

## Discussion

Responses to the open-ended question that discussed the impact of deployment on families were anticipated as the aim of the survey was to determine such impacts via validated quantitative measures. Additionally, many responses expanded on issues examined in the survey, namely work/family conflict, family functioning, coping styles, social support and use of services, attitudes to the military and the emotions and behaviours of children. However, research feedback, content for further research and issues of importance to respondents that were not covered in the survey were also obtained.

Expectations of support, particularly during deployment, corresponds with prior quantitative findings [1], [13]. The type and variety of support available to spouses has changed over time. Defence-associated and independent agencies provide deployment-related support services to families. These services include preparedness sessions and resources, courtesy calls, referrals and counselling [23]. Support for military life in general includes programs designed to build resilience in families, practical supports for spouse employment and entitlements for families living apart from the member [23]. The extent to which support resources are consistent and known, used and appropriate, and helpful to families should be continually evaluated and adapted as the needs of families evolve. The open-ended question responses suggest that consistency, ‘life course’ support and expectation management should be key goals of supporting services.

Responses about postings and non-deployment separations demonstrate that deployments are not the main or sole challenge for many spouses. Moving with postings has been negatively related to Army satisfaction in spouses, but not related to physical or psychological wellbeing [24]. The open-ended question responses may contradict the latter finding as respondents noted impacts on their wellbeing from missing family and challenges building long-term friendships. Regarding separations, increased time separated from the military member because of work demands has been significantly associated with lower spouse psychological wellbeing [25]. The open-ended question respondents particularly noted the ramifications of separation from the military parent on their children – an outcome likely to impact the wellbeing of spouses. More respondents discussed adverse impacts for their children than for themselves, suggesting that strategies to improve outcomes for children may improve spouse wellbeing.

The language and tone used by the spouses to convey the adverse influence of military life on their own employment was such that these responses were recalled most by the authors over other topics of response. This frustration for many spouses is borne out in quantitative research, with over half of the approximately 5,000 spouses in a survey on evaluations of the Australian Defence Force reporting that they had made employment or career sacrifices because of the military member's work [1]. Refinement of existing supportive policies may improve employment experiences for some spouses; however, as intimated by the respondents to the open-ended question, the nature of postings and negotiation within the spousal relationship will remain known challenges of military life. Given that spouse employment appeared in ‘negative experiences of military life' and ‘comments on the survey’ responses it is clear that this is an important issue for spouses that warrants further research.

The greater frequency of negative responses compared to other types of responses could represent a negativity bias found in open-ended question responses [18], [26]. A negativity bias exists in evaluation of specific events because negative stimuli have a higher affective impact than positive stimuli [27]. The survey participants and other respondents may have had positive or neutral experiences of support, deployment, postings or separation from the military member, for example, but did not express these in a response to the open-ended question. However, while approximately a fifth of respondents only provided a negative response, the other four fifths also provided other types of responses.

The positives, appraisals and coping responses provide spouses of military members with examples for dealing with military life challenges and framing their own experiences. In healthcare decision-making, people have been found to use other people's experiences to recognise decisions that need consideration, identify and appraise options and to support coping strategies [28]. Spouses of military members should be encouraged and supported to seek out the experiences of other spouses. Younger spouses have reported more military work/family conflict than older spouses [29], which could reflect a lack of experience or expectation management [30]. Satisfaction with military life for spouses of military members has been associated with perceived high levels of social support, internal locus of control, the temperament of adaptability, positive spouse employment experiences and marital quality [11]. Resilience programs offered by support agencies should be promoted and adequately resourced.

An open-ended question in a survey that was administered to spouses of serving, ex-serving, regular and reserve military members from each Service enabled qualitative data to be obtained from spouses from across the military spectrum. The broad nature of the question (‘anything else’) enabled respondents to discuss any aspects of their military life experiences, with most respondents discussing a range of topics within their response. The limitation of the investigation is that of all analyses of open-ended question responses – the inability to determine the reasons why people do or do not respond. Survey participants may not respond to an open-ended question due to lack of time, not having strong views or being inarticulate [18]. The finding that respondents had a greater level of education than non-respondents supports the latter possibility.

## Conclusions

This investigation obtained rich detail on the experiences of spouses of military members, particularly identifying key impacts on wellbeing, resilience strategies and ways support for families could be improved. The findings suggest that research on spouses of military members should examine multiple indicators of wellbeing, particularly spouse's satisfaction with their own employment. They also suggest that supporting agencies need to ensure appropriate support for different types of families and support that adapts over the life course. Spouses of military members may benefit from reading others' experiences, in terms of considering coping strategies and in framing their experience as a member of this unique population group.
