# Correction: Spouses of Military Members’ Experiences and Insights: Qualitative Analysis of Responses to an Open-Ended Question in a Survey of Health and Wellbeing

**DOI:** 10.1371/journal.pone.0118433

**Published:** 2015-02-06

**Authors:** The PLOS ONE Staff

There are errors in the Funding section. The correct funding information is as follows: Financial support for this particular investigation derived from the Timor-Leste Family Study dataset was provided by the School of Population Health, The University of Queensland. This work has been produced with the assistance of funding provided by the Department of Veterans’ Affairs. However, the views expressed in this version of the Work do not necessarily represent the views of the Minister for Veterans’ Affairs or the Department of Veterans’ Affairs. The Commonwealth does not give any warranty nor accept any liability in relation to the contents of this Work. The Department of Veterans’ Affairs had no role in study design, analysis, decision to publish, or preparation of the manuscript for this particular investigation.
